# Supplementary material for: The Glyoxalase System Is a Novel Cargo of Amniotic Fluid Stem-Cell-Derived Extracellular Vesicles
Source: Antioxidants (Basel). 2022 Aug 5;11(8):1524. doi: 10.3390/antiox11081524 (PMC9405222; doi:10.3390/antiox11081524)
Supplement: Supplementary file 1 [file antioxidants-11-01524-s001.zip › antioxidants-1816547-supplementary.pdf]

Supplementary Material: The glyoxalase system is a novel cargo of amniotic fluid stem cell-derived extracellular vesicles

Rita Romani, Vincenzo N. Talesa and Cinzia Antognelli

Figure S1. Whole blots reported in Figure 1a, Figure 3a, Figure 4, Figure 5a.

Figure 1a

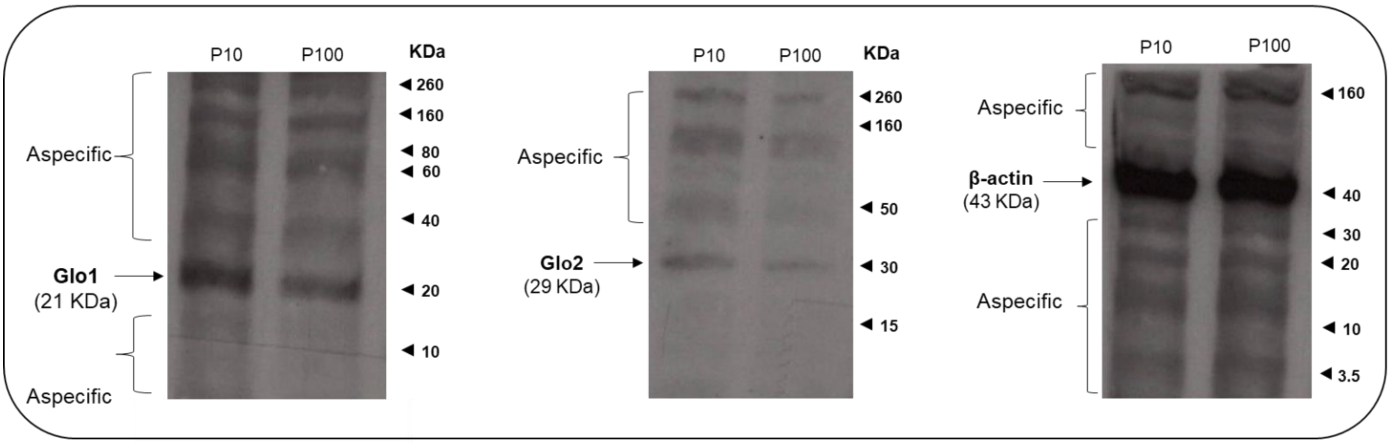

Figure 3a

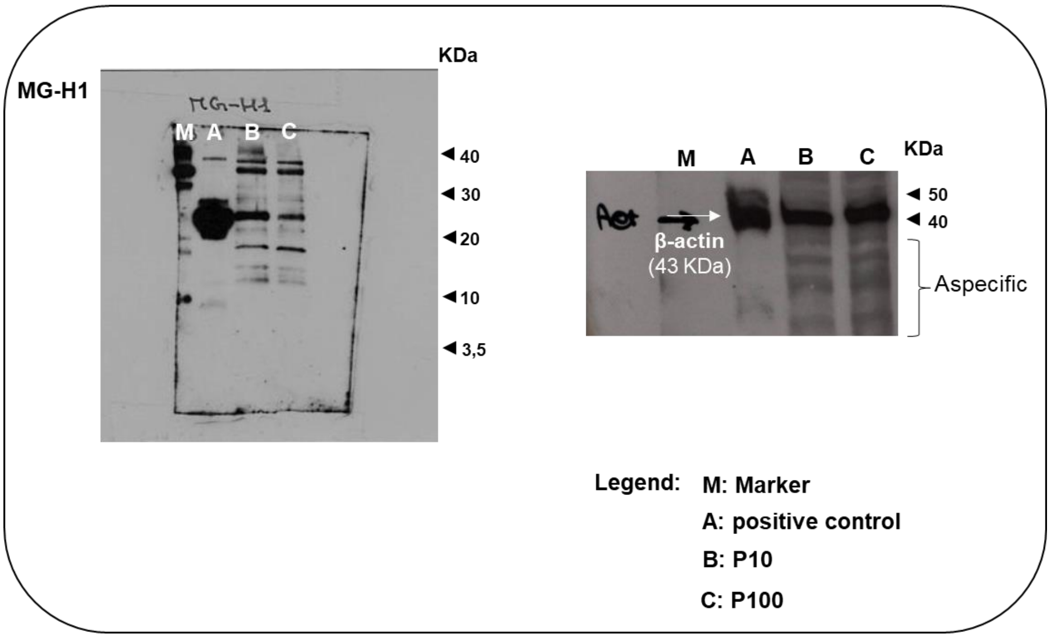

Figure 4

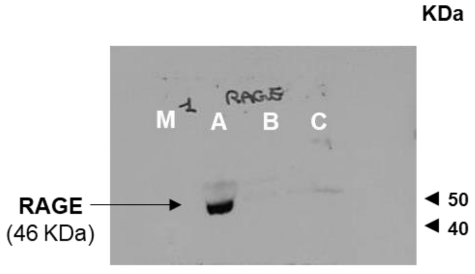

Legend: M: Marker
A: positive control
B: P10
C: P100

Figure 5a

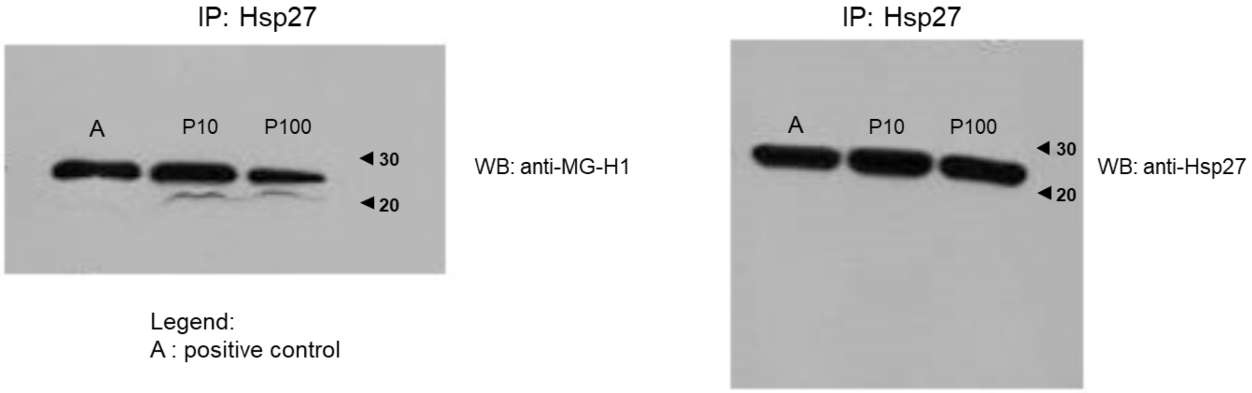

Legend:
A : positive control

Legend:
A : positive control
